# Circular utilization of discarded oyster farming bamboo scaffolding in pulp and papermaking

**DOI:** 10.1038/s41598-023-48191-5

**Published:** 2023-12-01

**Authors:** Hao-Chen Sun, Yu-Hsun Lai, Kuan-Yeh Huang, Ssu-Yu Huang, Jiann-Gwo Shyu, Yuan-Shing Perng

**Affiliations:** 1 Department of Forestry, National Chung Hsing University, Taichung, 40227 Taiwan, ROC; 2 Division of Polymer Research, Industrial Technology Research Institute, Hsinchu, 300044 Taiwan, ROC; 3 Forest Products Utilization Division, Taiwan Forestry Research Institute, Taipei, 100051 Taiwan, ROC

**Keywords:** Environmental sciences, Materials science

## Abstract

Oyster Farming is one of important fisheries and aquaculture industries in Taiwan. Each year, approximately 4000–5000 tons of discarded bamboo scaffolding (BS) used in oyster farming, are generated, so the treatment and utilization of BS should be taken seriously. This study evaluates the suitability of BS for pulp and papermaking by assessing the chemical compositions, microstructural, and fiber morphology. The pulping properties is investigated by soda pulping. The chemical composition of BS shows the potential for application in pulping. The BS microstructure shows that can enhance pulping reactions, while the fiber morphology indicates the possibility of producing high-strength paper. Through the pulping experiment, it demonstrated that BS is suitable for pulping with lower NaOH dosage and longer digestion time. The condition at 170 °C with 14% NaOH dosage for 90 min digestion has the highest yield. After refining the highest pulping yield BS pulp, it can improve the handsheet strength and bulk of the OCC-BS mixed pulp, which can achieve the strength property required for industrial paper. In summary, BS exhibits the potential for pulping application and produces a better paper strength than OCC pulp, exhibiting the feasibility of enhancing the circular utilization value of BS in Taiwan.

## Introduction

Oyster farming is one of the important fisheries and aquaculture industries in Taiwan. The cultivation regions primarily extend along the coastal areas of Hsinchu, Changhua, Yunlin, Chiayi, and Tainan. The methods of the oyster farming include bamboo rafts, hanging, suspension, and long-line methods, with the floating raft culture method being predominantly utilized in the Tainan area^1,2^. The floating raft is usually constructed with 43 bamboo poles, including 3 to 4-year-old Thorny, Makino, or Long-branch bamboo of 34 poles with a length of 9 m, and 4-year-old Moso bamboo of nine poles with a length of 8 m. Hence, the rafts are also referred to as bamboo scaffolding. The service life of bamboo scaffolding is approximately two to three years. After its use, the discarded oyster farming bamboo scaffolding (BS) needs to be managed and recycled. Almost 4000 to 5000 tons of BS are generated each year^3^.

In recent years, the circular utilization of agricultural waste has gained increasing attention. It can serve as raw material for various purposes, producing high-value-added products^4,5^. Additionally, this utilization can be referred to as the utilization of agricultural surplus materials. Nowadays, BS can be utilized in producing biocarbon and wood vinegar, but the quantity of production cannot cover the recycled amounts. Part of BS is breaking down to manufacture the fuel rods by specific company. However, during the processing, the government needs to spend USD 158.04 to 189.65 per ton annually^2^. Therefore, after the recycling of BS, there is still a need to develop more economically viable and suitable methods of circular utilization. Ho and Lin^6^ analyzed the chemical compositions of BS, and the result shows a holocellulose content of 75.06%, α-cellulose content of 49.40%, and lignin content of 26.31% in BS, which is similar to native bamboo. In this case, BS may have a potential to be developed as raw materials for value-added fiber applications, thus enhancing its economic value.

As the paper industry advances, the global demand for paper and paperboard is progressively increasing each year^7^, indicating a growing need for feedstock in papermaking, which becomes imperative to explore novel raw materials in response to the increasing demands in production. In Taiwan, industrial paper is the primary product of the papermaking industry, with its production accounting for 82.0% of the total in 2021, which is also increasing annually^8^. According to the Taiwanese papermaking industry situation, the recycled paper utilization ratio is 97.9%, the primary feedstock is the old corrugated cardboard (OCC) pulp, but 34.2% of this material is imported. The increasing production exhibits the growing demand for fiber feedstocks, which means the agricultural surplus fiber materials have an opportunity to be applied in industrial paper production according to the Taiwanese papermaking industry trend, and the development of novel materials may have a chance to reduce dependence on imported materials of the Taiwanese papermaking industry. The agricultural surplus fiber materials have been studied and applicated a lot in pulping and papermaking, like rice straw or wheat straw pulping^9,10^, but the surplus fiber materials of aquaculture or fisheries, like the wooden boat, bamboo raft, bamboo scaffolding for fisheries, are seldom discussed. These surplus materials still have a circularly utilized potential in fibrous value-added applications.

This study aims to utilize BS in pulp and papermaking, not only providing a novel material for the Taiwanese papermaking industry, but also enhancing the value of BS circular utilization. There is currently no relevant research on pulping and papermaking regarding BS materials, but its raw material is bamboo, so the pulping conditions and methods of this study is designed with reference to bamboo pulping. Chang et al.^11^ conducted pulping of 0.2, 1.2, and 3-year-old Thorny bamboo using kraft and soda methods. For kraft pulping, active alkali (AA) dosage of 16%, 18%, and 20% were used, along with a sulfidity of 25% and a digestion time of 90 min. For soda pulping, AA dosage of 16%, 18%, and 20% were used, with a digestion time of 150 min. Both pulping methods maintained a solid-to-liquid ratio of 1/4, a digestion temperature of 160 °C, and a heating-up time of 90 min. Among these, the 3-year-old Thorny bamboo, which shared the same source as BS raw materials, achieved the highest pulp yield of 40.37% under kraft pulping conditions of 20% AA dosage. For soda pulping, the highest pulp yield was 38.70% under conditions of 16% AA dosage. Mohamad Khair and Masrol^12^ conducted pulping of 3-year-old *Dendrocalamus asper* using soda methods, with the conditions of 25% of AA dosage, a solid-to-liquid ratio of 1/7, heating-up time of 70 min, digestion temperature of 170℃ for digesting 3 h. After digestion, the pulping yield is 20.88%. Based on the above studies, research exists regarding the application of soda and kraft methods in bamboo chemical pulping, with the typical AA dosage of 14–21% within pulping conditions. Among the two pulping processes, soda pulping of agricultural surplus materials has been industrially adopted in some countries^13–15^. Consequently, this research opts for the soda methods to assess the pulping properties of BS.

The purpose of this study is to investigate the feasibility of utilizing BS in pulping and papermaking applications, with the goal of enhancing the circular utilized value of BS. The study evaluates the pulping and papermaking potentials of BS by the chemical compositions, microstructural appearance and fiber morphology analysis. Simultaneously, the original materials (4-year-old Moso bamboo and 3-year-old Thorny bamboo) used to construct BS are act as control groups to analyze material properties and explore changes in BS properties due to seawater immersion. The soda pulping experiment for BS employs a factorial experimental design to investigate how digestion time and AA dosage impact BS pulp properties, including pulping yield (shives, accepts, fines content), pulp freeness, and handsheet physical properties (tensile, burst, ring crush index, bulk). After the pulping experiments, the sample with the highest pulping yield is blended with TOCC (Taiwan OCC) in various ratios to examine changes in freeness and handsheet properties. This aims to assess the feasibility of applying BS pulp in industrial paper manufacturing, which means the BS pulp needs to achieve the property level of OCC pulp (primary feedstock of industrial paper) to have an opportunity to be utilized. The study hopes to develop a novel circular utilization approach for BS.

## Experimental

### Materials

Discarded oyster farming bamboo scaffolding (BS) which is already been dried and chopped is provided by Tainan City Fishing Harbor and Coastal Fisheries Management Office (Tainan, Taiwan), with a moisture content of 9.21%. 4-year-old Moso bamboo (MB) (*Phyllostachys pubescens*) is harvested from Zhushan, Nantou, Taiwan, and dried by air for two weeks before chopping, and the moisture content is 14.54%. 3-year-old Thorny bamboo (TB) (*Bumbusa stenostachia* Hackel) is provided by National Pingtung University of Science and Technology (Pingtung, Taiwan), it is also dried by air for two weeks before chopping, the moisture content is 17.23%. After chopping, all the materials are selected out of the small chips with a length of 0.5–1 cm, width of 0.5–1 cm, and thickness of 0.3–0.5 cm, prepared for pulping experiments. The TOCC and JOCC pulps are provided by Houli Mill, CHENG LOONG Co., Ltd. (Taichung, Taiwan), with freeness of 385 mL and 405 mL and pulp consistency of 39.23% and 37.66%, respectively.

## Method

**Figure 1:**
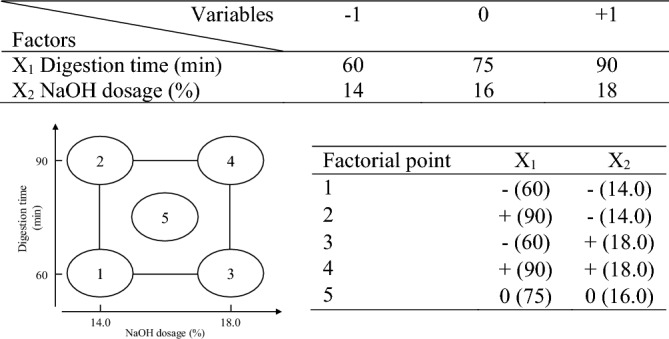
Factorial experiment design of BS soda pulping.

The constant conditions for BS soda pulping experiment include the digestion temperature of 170 ℃, the solid-to-liquid ratio of 1:5, the heating rate of 1.5 ℃/min, and BS weight of 500 g (oven-dried weight, od). The solid-to-liquid ratio is different from the bamboo pulping ^11^, because it is found that the lower solid content during the prepared experiment results in uneven digestion. The variables of the factorial experiment design are shown in Fig. 1, including the digestion time (X_1_) with a high level (+ 1) of 90 min and low level (− 1) of 60 min, and the NaOH dosage (X_2_) with high level (+ 1) of 18% and low level (− 1) of 14%. In the meantime, there is a middle point (0) with a digestion time of 75 min and a NaOH dosage of 16%. In the pulping process, the additional added water and NaOH dosage are calculated first, and then the additional added water is used to soak BS chips. After soaking, the NaOH is mixed with the chips and placed into the rotary digester (Model 8060, Lesson Industrial Co., Ltd., New Taipei, Taiwan). Then, the pulping process begins, following the condition mentioned above. After digestion and cooling, the pulp is washed under tap water using the 200 mesh sieve until the filtered water becomes colorless. When the pulping process is finished, the pulp is placed into a plastic bag in preparation for analysis.

## Material properties

### (1) Chemical compositions

The materials are pulverized by the high-speed pulverizing machine (RT-02B, Rong Tsong Precision Technology Co., Ltd., Taichung, Taiwan), and the particles between 40 and  60 meshes are screened out for chemical composition analysis, including ash (TAPPI T 211 om-22), alcohol-benzene extractives (TAPPI T 204 cm-17), holocellulose (ASTM D1104-56), acid-insoluble lignin (TAPPI T 222 om-21), and α-cellulose (TAPPI T 203 cm-22), each analysis is repeated twice to calculate the average and standard deviation.

### (2) SEM–EDS

The microstructural appearance of BS is observed using a scanning electron microscope (SEM, Zeiss Ultra Plus, Zeiss, Germany). The operating voltage during the observation is set at 15 kV. Simultaneously, surface elemental compositions are assessed through Energy-dispersive X-ray spectroscopy (EDS, OXFORD X-Max 50 mm^2^, Oxford Instruments, UK) to determine the presence of any metal elements deposited on BS surface from the sea. A comparative analysis is conducted between BS and reference samples MB and TB.

### (3) Fiber morphology

The method of fiber morphological analysis is according to Franklin’s method^16^. Initially, the materials are immersed in a digestion solution comprising one part hydrogen peroxide, five parts glacial acetic acid, and four parts distilled water. This mixture is allowed to react at 40 ℃ for a period of 7 days to disintegrate the fiber structure. After reaction, the white and transparent fiber is measured by fiber image analyzer (FS5, Valmet Co., Ltd., Finland), and recording fiber length (mm), fiber width (μm), cell wall thickness (μm), and coarseness (mg/m). Otherwise, the L/D value (slender ratio) is calculated by the length to width ratio. All the samples are conducted with six times to record the average and standard deviation.

## Pulp properties

### (1) Pulping yield

After pulping, the sample is screened using the flat screen (Model 302, Lesson Industrial Co., Ltd., New Taipei, Taiwan) with the slot width of 0.25 mm according to Tappi UM 204. In the unscreened pulp, the material unable to pass through the 0.25 mm sieve is termed Shives, while that passing through and collected by a 200 mesh sieve is referred to as Accepts. The lost material is collectively termed Fines. After screening, Collective Shives and Accepts are measured the total oven-dried weight and determine Shives, Accepts, and Fines ratios using Eqs. (1), (2), and (3) respectively.

1 $$\mathrm{Shives\,\, ratio }\left(\mathrm{\%}\right)=\frac{Shives\,\, oven\,\, dried\,\, weight (g)}{Raw \,\,material\,\, oven \,\,dried \,\,weight (g)}\times 100$$

2 $$\mathrm{Accepts\,\, ratio }\left(\mathrm{\%}\right)=\frac{Accepts\,\, oven\,\, dried\,\, weight (g)}{Raw \,\,material \,\,oven \,\,dried \,\,weight (g)}\times 100$$

3 $$\mathrm{Fines \,\,ratio }\left(\mathrm{\%}\right)=100-\mathrm{Shives\,\, ratio }\left(\mathrm{\%}\right)-\mathrm{Accepts \,\,ratio }(\mathrm{\%})$$

### (2) Pulp freeness

The measurement of pulp freeness is conducted using a Canadian standard freeness (CSF) tester (Model 305, Lesson Industrial Co., Ltd., New Taipei, Taiwan), according to TAPPI T 227 om-17. Each sample is tested twice, and the average result is recorded.

### (3) Accepted fibers appearance

The accepted fiber appearance is observed using the optical microscope (Eclipse E100, Nikon, Japan), and during the experiment, the pulp concentration is adjusted to 0.4% to disperse the fibers for specimen preparation. The images of the fibers are captured by the microscope camera (HDC588, MicroTech, Canada) using the MicroCam V5 software (M&T Optics Co., Ltd., Taipei).

### (4) Handsheet properties

After soda pulping, handsheets were prepared for each factorial point in Fig. 1 to analyze the impact of pulping conditions on handsheet properties. Simultaneously, the sample with the highest pulping yield are refined by the 10 L Hollander beater (Model 304, Lesson Industrial Co., Ltd., Taiwan) according to TAPPI T 200 sp-21 for 60 min to adjust the freeness. After refining, BS pulp is blend with TOCC at BS to TOCC ratio of 100/0, 50/50, 0/100. The freeness and handsheet properties are then analyzed. The handsheet preparation is according to TAPPI T 205 sp-12, the grammage is set at 100 g/m^2^ to simulate the grammage of cardboard. After the handsheet making, the handsheet is conditioned under 50.0% ± 2.0% RH and 23.0 ± 1.0 °C for at least 4 h according to TAPPI T 402 sp-13 before testing. The grammage and thickness of the handsheet were measured according to TAPPI T 410 om-08 and T 411 om-21, respectively, then the handsheet bulk was calculated. Six handsheets with similar grammage and intact appearance were screened for strength properties testing. The testing items were mainly the paper properties specified by Taiwan CNS standards for cardboard paper, including tensile index (TAPPI T 494 om-01), burst index (TAPPI T 403 om-15), ring crush index (TAPPI T 818 cm-18). The experimental results are recorded by calculating the average value and standard deviation of six test data.

### Statistical analysis

The process of factorial design analysis follows the guidelines outlined in 'Design and Analysis of Experiments 7/e' by Montgomery^17^, using Microsoft Excel (Microsoft, USA) to analyze. The result of handsheet properties, BS pulp blend into TOCC, are analyzed using IBM SPSS Statistics 20 (IBM, USA) for one-way analysis of variance (ANOVA). The Turkey Honestly Significant Difference (HSD) test was employed for multiple comparisons, employing a 95% confidence interval. A p-value < 0.05 indicated statistical significance.

## Results and discussion

### Material properties

#### (1) Chemical compositions

The chemical compositions of BS are shown in Table 1, with an ash content of 2.12%, a toluene-alcohol extractives content of 2.76%, a holocellulose content of 68.04%, an acid-insoluble lignin content of 25.03%, and an α-cellulose content of 49.07%. In comparison with the research by Ho and Lin^6^, this study showcased lower ash content in BS, similar levels of toluene-alcohol extractives and acid-insoluble lignin content, a relatively lower holocellulose content, and a higher α-cellulose content. The composition variation in BS could be attributed to the diversity of bamboo materials constructed, as well as variations in usage conditions and climate, leading to differences in service life and the final compositions.

**Table 1:** Chemical compositions of BS.

References	Ash (%)	Toluene-alcohol extractives (%)	Holocellulose (%)	Acid-insoluble lignin (%)	α-cellulose (%)
This study	2.12 ± 0.12	2.76 ± 0.27	68.04 ± 3.57	25.03 ± 0.98	49.07 ± 1.18
Ho and Lin (2020)	11.19 ± 1.48	3.24 ± 0.93	75.06 ± 0.80	26.31 ± 0.16	39.40 ± 1.06

The chemical compositions of BS, native bamboo (MB, TB), and other fiber materials used in pulping are presented in Table 2. The ash content of BS is higher than MB but lower than TB (this study; Chang et al.^11^), suggesting that seawater immersion did not significantly increase the ash content. The holocellulose content of BS is similar to native bamboo, softwood, and hardwood. The acid-insoluble lignin content is higher than rice straw, wheat straw, and hardwood, but similar to native bamboo, and lower than softwood. Meanwhile, the α-cellulose content is similar to TB and higher than in other materials. In summary, the chemical composition of BS suggests its potential for application in pulping.

**Table 2:** Chemical composition of BS and other fiber materials.

Materials	Ash (%)	Toluene-alcohol extractives (%)	Holocellulose (%)	Lignin (%)	α-cellulose (%)	References
BS	2.12 ± 0.12	2.76 ± 0.27	68.04 ± 3.57	25.03 ± 0.98	49.07 ± 1.18	This study
MB	0.78 ± 0.09	3.90 ± 0.39	65.67 ± 4.65	27.27 ± 0.92	45.36 ± 0.88	This study
TB	2.25 ± 0.67	2.98 ± 0.95	63.08 ± 1.19	23.97 ± 0.26	50.80 ± 0.34	This study
TB	2.66 ± 0.028	4.75 ± 0.141	68.53 ± 0.11	25.21 ± 0.057	–	^11^
Rice straw	15.8	1.7	59.0	21.0	31.1	^9^
Wheat straw	7.15 ± 0.77	3.07 ± 0.24	–	13.91 ± 0.52	39.36 ± 0.64	^22^
Softwood	–	–	65–74	25–31	40–45	^23^
Hardwood	–	–	67–82	16–24	43–47	

*BS* oyster farming bamboo scaffolding, *MB* 4 year-old Moso bamboo, *TB* 3 year-old Thorny bamboo.

#### (2) Material appearances

**Figure 2:**
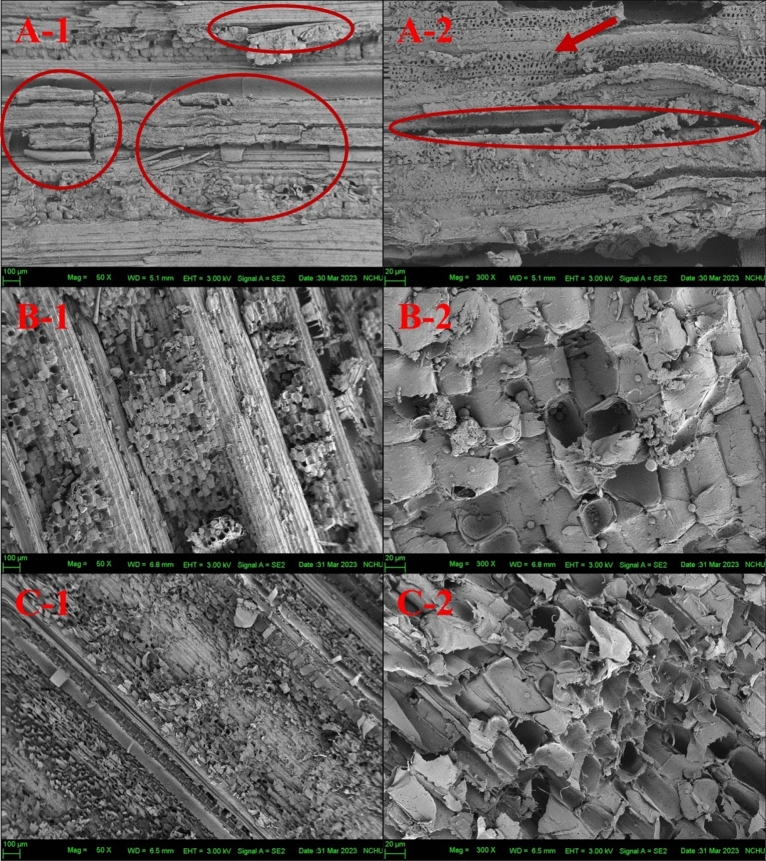
SEM images of (**A**) BS, (**B**) MB and (**C**) TB (Code 1 and 2 are associated with ×50 and ×300 magnification, respectively).

To figure out the effect of seawater immersion on the fibrous structure of BS, SEM is used for the observation of microstructure on BS to compare with MB and TB, SEM image is showed in Fig. 2. By analyzing the microstructure images at a magnification of 50×, it is evident that the surface structure of BS (Fig. 2A-1) appears relatively looser in arrangement compared to that of MB (Fig. 2B-1) and TB (Fig. 2C-1). On the surface of BS, numerous cracks perpendicular to the longitudinal direction and fracture structures are observed, as indicated by the red circles. As the magnification is raised to 300×, the surface of BS (Fig. 2A-2) reveals a significant number of pores, indicated by the red arrows, whereas the native bamboo (Fig. 2B-2, C-2) are less pronounced and noticeably fewer. This formation of pores may be attributed to the erosion from seawater and weathering that BS undergoes during usage, resulting in the structure. These pores, referred to as mesoporous pores, can increase the surface roughness of the material and enhance the permeability of chemicals during the pulping process^18^. Therefore, it can be inferred that BS may be more susceptible to penetration compared to native bamboo during pulping.

#### (3) Surface elements

**Figure 3:**
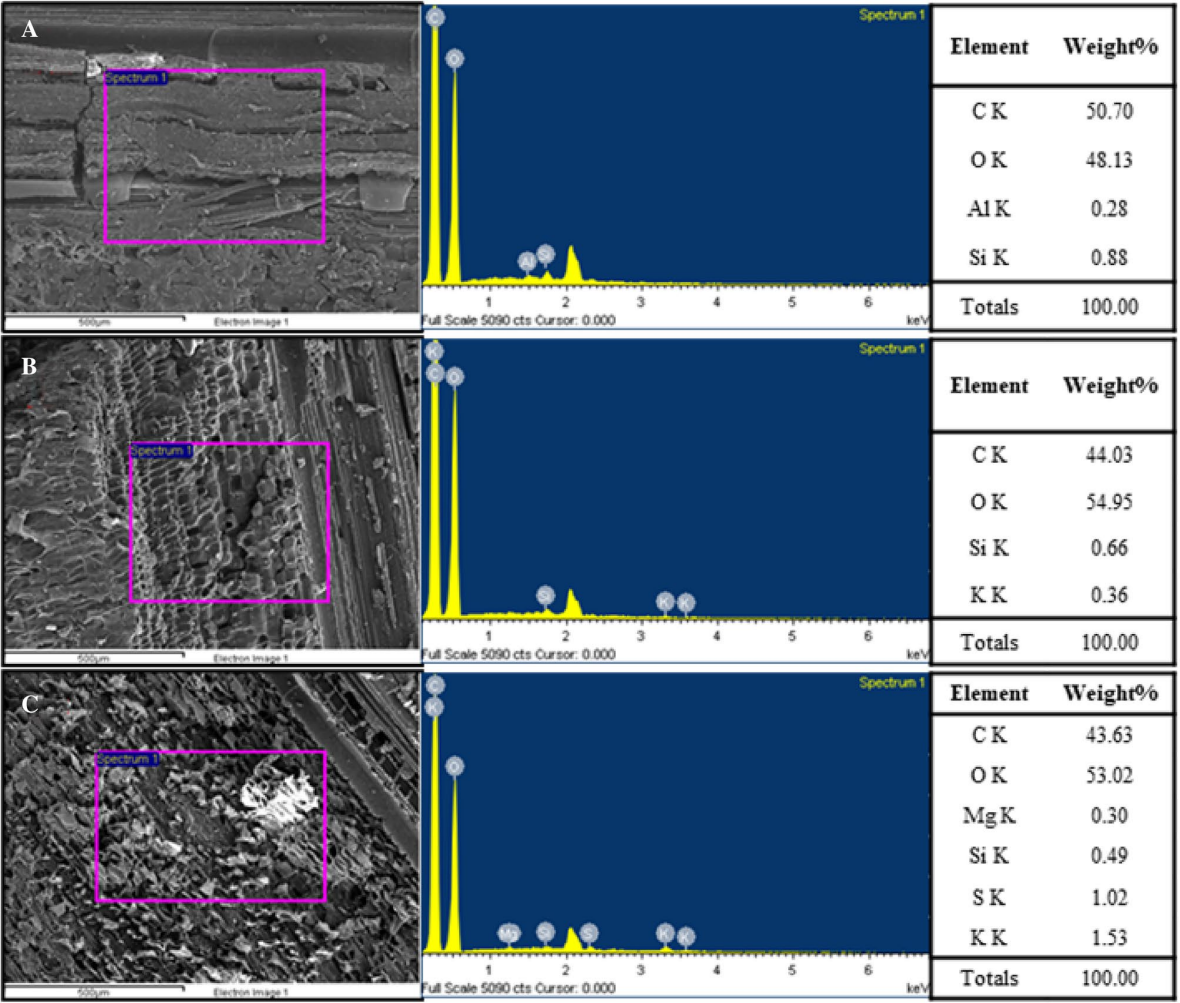
EDS analysis of (**A**) BS, (**B**) MB and (**C**) TB.

To validate the lower ash content result in BS, this study employed EDS analysis to examine the surface elemental composition of BS, in order to confirm the presence of salt deposits from seawater on the surface. The analytical results are presented in Fig. 3. The content and types of metal element of the surface of BS (Fig. 3A) are not even higher than native bamboos (Fig. 3B, C). The content and types of metal elements on the surface of BS (Fig. 3A) are not even higher than native bamboo (Fig. 3B, C). However, the BS material in Ho and Lin's study^6^ revealed the results of EDS analysis, indicating element content of 2.08% Na, 1.49% Si, 3.72% Cl, and 6.76% Ca. These findings suggest a higher content of sea salt elements on the surface. But the ash content (2.12%) in the BS of this study is significantly lower than that in the study (11.19%) by Ho and Lin^6^ It can be seen that the BS used in this study has a lower degree of salt deposition, and the ash content is similar to the native bamboo, which indicates that BS of this study is suitable for pulping.

#### (4) Fiber morphology

The paper properties are influenced by the fiber morphology of the pulp. Therefore, this study evaluates the fiber morphology of BS, and compares with MB and TB, after defibration using Franklin's method, in order to assess the feasibility of utilizing the BS fibers for papermaking. The results are presented in Table 3. The fiber morphology of BS includes the fiber length (Fl) of 1.996 mm, fiber width (Fw) of 19.08 μm, cell wall thickness (CWT) of 4.93 μm, and the coarseness of 0.028 mg/m, simultaneously, the L/D value is 104.63 calculated from length-to-width ratio. To compare with other materials, the Fl of BS is between MB (1.991 mm) and TB (2.403 mm), similar to Birch (1.8 mm) and the average of hardwood (2.0 mm), but it is lower than the bamboo (2.8 mm) recorded by Smook^19^. The Fw of BS is closed to native bamboo (MB: 18.27 mm,TB: 19.01 mm), and lower than softwood and hardwood. The CWT of BS is thicker, but the coarseness is the lowest in Table 3, which can indicate that the density of BS CWT is lower and more prone to flattening, thereby increasing the contact area between fibers of the paper structure^19,20^. The L/D value of BS is lower than native bamboo but higher than wood. El Omari et al.^21^ suggest that fiber with an L/D value > 70 can produce high-strength properties paper. In summary, the fiber morphology of BS has the potential for application in papermaking.

**Table 3:** Fiber morphology of BS and other fiber materials.

Materials	Fl (mm)	Fw (μm)	CWT (μm)	Coarseness (mg/m)	L/D	References
BS	1.996 ± 0.040	19.08 ± 0.35	4.93 ± 1.77	0.028 ± 0.005	104.63 ± 1.15	This study
MB	1.991 ± 0.014	18.27 ± 0.20	3.14 ± 0.36	0.149 ± 0.012	108.98 ± 1.59	
TB	2.403 ± 0.020	19.01 ± 0.29	0.96 ± 0.04	0.054 ± 0.002	126.41 ± 2.91	
Bamboos	2.8	15	–	–	180	^19^
Birch	1.8	20–36	3–4	0.05–0.08	50–90	
Hardwood	2.0	22	–	–	90	
Soft wood	4.0	40	–	–	100	

*BS* bamboo scaffolding, *MB* 4 year-old Moso bamboo, *TB* 3 year-old Thorny bamboo, *Fl* fiber length, *Fw* fiber width, *CWT* cell wall thickness, *L/D* length/width ratio (slenderness ratio).

### Pulp properties

#### (1) Pulping yield

The factorial analysis of pulping yield is presented in Table 4. The accepts ratio is significantly affected by the factor of X_1_X_2_ interaction, the value is -3.940. The shives ratio is significantly affected by all factors, the absolute values from high to low are X_2_ |-10.265|> X_1_X_2_ |1.735|> X_1_ |-1.715|, which indicates X_2_ has more impact on shives ratio. The fines ratio is significantly affected by X_2_, the value is 10.175.

**Table 4:** Factorial analysis of pulping yield.

Factor	Accepts ratio (%)	Shives ratio (%)	Fines ratio (%)
X_1_	0.640	−1.715*	1.105
X_2_	0.120	−10.265*	10.175*
X_1_X_2_ interaction	−3.940*	1.735*	2.235
Assessed value	1.438	0.629	8.895
Pooled standard deviation (s)	0.113	0.049	0.700

*The factor impact is significant; factor X_1_: digestion time; factor X_2_: NaOH dosage.

**Figure 4:**
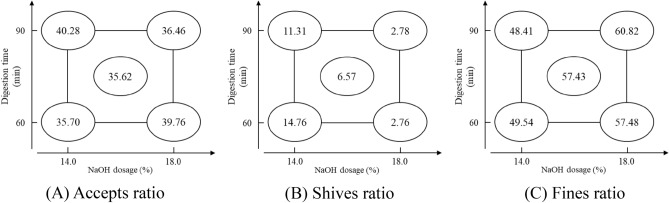
Factorial analysis chart of pulping yield.

The factorial analysis charts of pulping yield are shown in Fig. 4. From the comprehensive comparison of the results of the analysis chart for (A) accepts ratio, (B) shives ratio, and (C) fines ratio in Fig. 4, it can be observed that the effect of digestion time becomes more pronounced at a NaOH dosage of 14%. With increasing time, the pulping yield is enhanced. However, it is more significantly influenced by NaOH dosage. As the NaOH dosage increases, the shives ratio decreases, while the fines ratio increases. Specifically, at lower digestion times, a higher NaOH dosage level corresponds to a higher accepts ratio. In contrast, with longer cooking times, an increased NaOH dosage leads to a heightened degree of fiber degradation, resulting in a decreased accepts ratio. To sum up, BS is suitable for soda pulping with lower NaOH dosage (14%) and higher digestion time (90 min), which has the highest accepts ratio of 40.28%. To compare with Chang et al.^11^, BS soda pulp demonstrates a higher acceptance ratio with lower NaOH dosage, shorter digestion time, and an even higher solid-to-liquid ratio, which can also be inferred as a pulping result of the lower concentration of NaOH solution and exhibiting the easier condition of the of pulping with BS.

#### (2) Accepted fiber appearances

**Figure 5:**
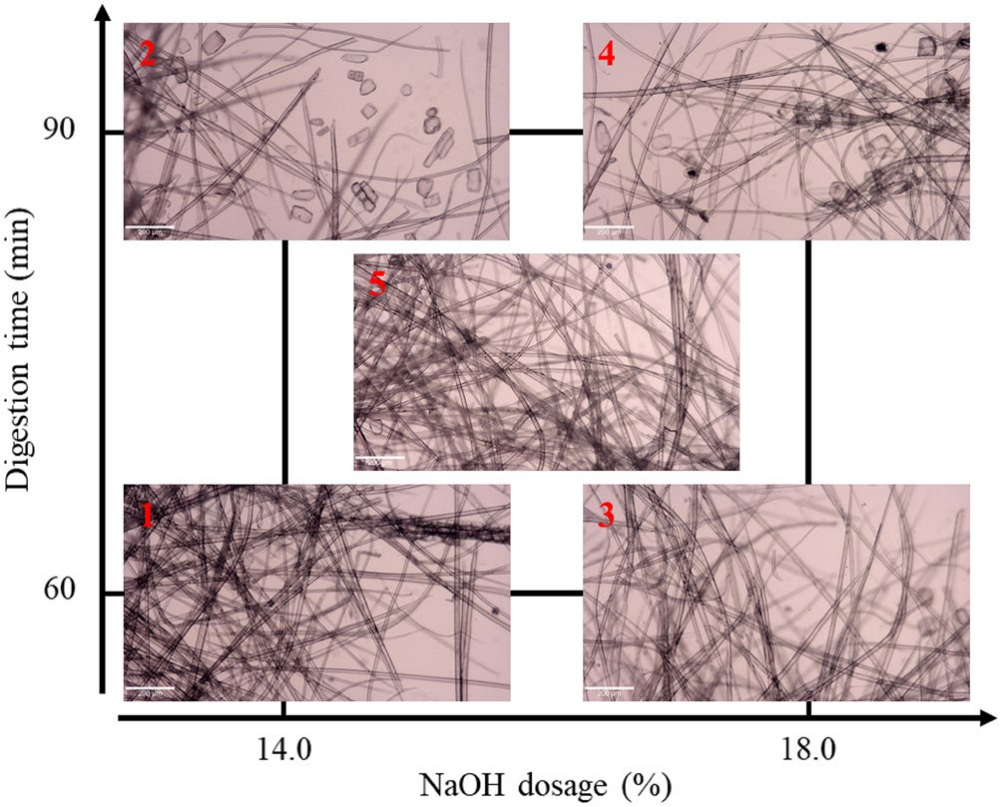
Optical microscope images of accepted fibers.

The accepted fibers are observed under the optical microscope, the images with the magnificent of 40× are shown in Fig. 5. The images are arranged in the factorial design chart, and it can be observed that the images of the factorial points are similar to each other. The fibers both exhibit a favorable state of dispersion and a slender shape. It can be indicated that the digesting conditions have no significant effect on fiber appearance, cause all fibers are similar. Simultaneously, the pulp has not undergone refinement, thus the fiber surface does not exhibit any fines or fibrillated fibers.

#### (3) Freeness

**Figure 6:**
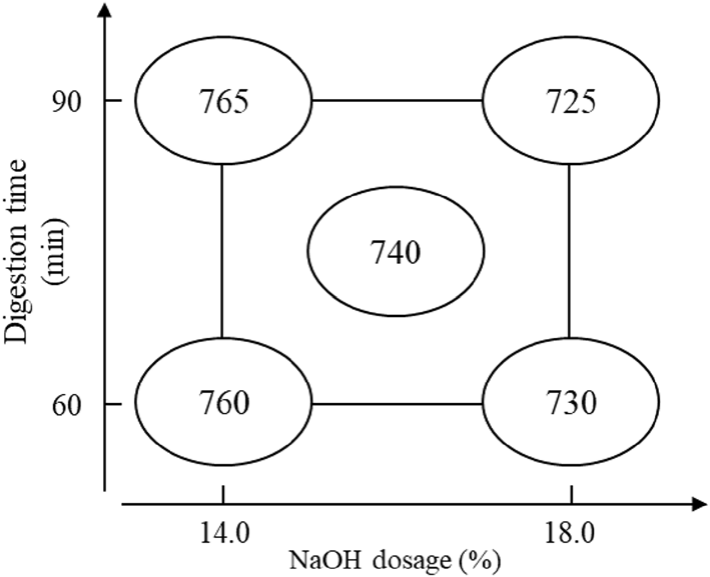
Factorial analysis chart of pulp freeness.

The accepted fibers freeness of the BS soda pulp is directly measured after screening, and the results of the factorial analysis are presented in Table 5. The factors of X_1_ and X_1_X_2_ interaction have a significant impact on freeness. The factorial analysis chart (Fig. 6) shows that the freeness is decreasing as well as NaOH dosage is increasing under the constant digestion time. Hence, it can be indicated that the NaOH dosage significantly influences the reduction in the freeness during the BS soda pulping process.

**Table 5:** Factorial analysis of pulp freeness.

Factor	CSF (mL)
X_1_	0.0
X_2_	−35.0*
X_1_X_2_ interaction	−5.0*
Assessed value	0.0
Pooled standard deviation (s)	0.0

*The factor impact is significant; factor X_1_: digestion time; factor X_2_: NaOH dosage.

#### Handsheet properties

**Figure 7:**
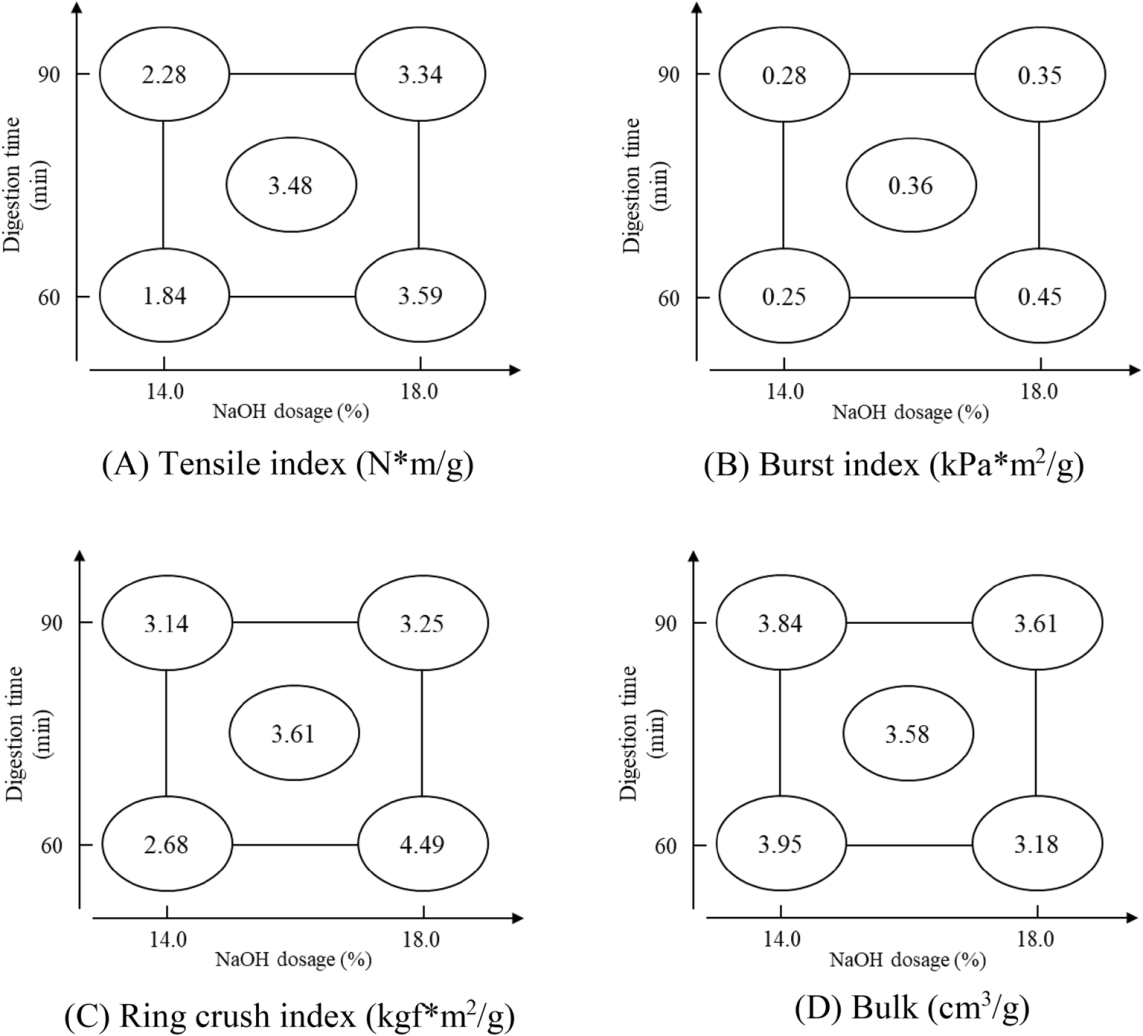
Factorial analysis chart of handsheet properties.

The factorial analysis of handsheet properties is presented in Table 6. The tensile and burst index are significantly affected by factor of X_2_, with effect values of 1.405 and 0.135, respectively. The ring crush index is significantly affected by X_2_ and the X_1_X_2_ interaction, with the absolute value comparison of the effect value as X_2_ |0.960|> X_1_X_2_ |−0.850|. The bulk is also affected by X_2_ and the X_1_X_2_ interaction, with the absolute value comparison of the effect value as X_2_ |−0.500|> X_1_X_2_ |0.270|. From the factorial analysis chart (Fig. 7), it is evident that increasing the NaOH dosage enhances the strength properties while reducing bulk. Additionally, the negative effect value of the X_1_X_2_ interaction implies that the condition with higher NaOH dosage and longer digestion time leads to poorer handsheet strength, which can be indicated that excessive fiber degradation (higher Fines ratio) and predominantly contributes to the decline in strength properties. None of the samples underwent refining, resulting in a freeness measurement exceeding 700 mL. Consequently, the handsheets displayed relatively diminished strength properties. To evaluate the feasibility of cardboard production, the BS pulp sample with the highest pulping yield (point 2, 90 min; 14%) is refined by Hollander beater for 60 min and blended with TOCC to test the freeness and handsheet properties.

**Table 6:** Factorial analysis of handsheet properties.

Factor	Tensile index (N*m/g)	Burst index (kPa*m^2^/g)	Ring crush index (kgf*m^2^/g)	Bulk (cm^3^/g)
X_1_	0.095	−0.035	−0.390	0.160*
X_2_	1.405*	0.135*	0.960*	−0.500*
X_1_X_2_ interaction	−0.345	−0.065	−0.850*	0.270*
Assessed value	0.449	0.090	0.449	0.090
Pooled standard deviation (s)	0.035	0.007	0.035	0.007

*Means the factor impact is significant; Factor X_1_: Digestion time; Factor X_2_: NaOH dosage.

The result of BS pulp blend with TOCC for papermaking experiment is presented in Table 7. After refining for 60 min, the freeness of BS pulp is decreased from 765 to 350 mL. As BS pulp is blended into TOCC, the freeness of mixed pulp is higher than TOCC, indicating that BS pulp can enhance the dewatering rate. The phenomenon could be attributed to the high slenderness ratio of BS fibers, resulting in more pores developing during the process of fiber accumulation. As a consequence, the water filtration property is improved. With the increase of the blending ratio of BS pulp in the mixture, the tensile, burst, and ring crush index of handsheets all increased, even reaching the strength level of TOCC and JOCC. Besides, as the blending ratio of BS pulp raised, the bulk of the handsheet increased, and both were significantly higher than TOCC and JOCC. The above result is obtained under handsheet making without any chemical addition. As shown in Table 7, some of the mixture samples can reach the basic strength quality specification of B-grade corrugating medium paper and C-grade liner board paper, which are presented as the main products of Taiwanese industrial papermaking, in Taiwan CNS standards. The increasing freeness, bulk, and strength properties correspond to the requirement of dewatering efficient, higher bulk in the specific strength properties in the papermaking industry. Therefore, BS can be utilized in industrial papermaking after pulping.

**Table 7:** Handsheet properties of BS soda pulp blended with TOCC.

Sample (BS: TOCC)		CSF (mL)	Tensile index (N m/g)	Burst index (kPa m^2^/g)	Ring crush index (kgf m^2^/g)	Bulk (cm^3^/g)
BS	TOCC					
100	0	350	43.40 ± 1.52^c^	2.54 ± 0.06^d^	12.86 ± 0.51^c^	2.08 ± 0.03^c^
50	50	395	30.15 ± 0.95^a^	1.68 ± 0.05^c^	10.66 ± 0.44^b^	2.00 ± 0.03^b^
0	100	385	28.62 ± 1.70^a^	1.03 ± 0.05^a^	9.29 ± 0.04^a^	1.79 ± 0.06^a^
JOCC		405	32.85 ± 2.063^b^	1.37 ± 0.09^b^	10.24 ± 0.32 ^b^	1.76 ± 0.03^a^
The quality requirement standard for corrugated board paper in CNS						
Corrugating medium (CNS 1455 B-grade)		–	> 29.43	–	> 7	–
Liner board (CNS 2955 C-grade)		–	–	2.0	> 10	–

Comparison of the data for different groups was performed by using Turkey’s HSD test. The significant level is set at p ≤ 0.05; Group a < b < c < d.

## Conclusion

The results of BS material properties present the potential for application in pulping and papermaking, including the higher α-cellulose content, the existence of mesoporous pores, and the fiber morphology suitable for papermaking. After the pulping experiment, the pulp properties (yield, freeness, handsheet) are mainly affected by the NaOH dosage. According to the results, the soda pulping condition of BS with lower NaOH dosage (14%) and higher digestion time (90 min) has the highest pulping yield. The BS pulp blended into TOCC can enhance the freeness, handsheet strength, and bulk properties, exhibiting the potential and feasibility of application in industrial paper production. In conclusion, the BS can be utilized as the feedstock of pulping and papermaking, providing a novel material and enhancing the value of BS in circular utilization.

## Data Availability

The datasets presented in this current study are available from the corresponding author on reasonable request.

## Abbreviations

BS: Discarded oyster farming bamboo scaffolding
MB: 4-Year-old Moso bamboo
TB: 3-Year-old Thorny bamboo
OCC: Old corrugated container
TOCC: Taiwan OCC
JOCC: Japan OCC
Fl: Fiber length
Fw: Fiber width
CWT: Cell wall thickness
